# Immunohistochemical evaluation of inflammatory and proliferative markers in adjacent normal thyroid tissue in patients undergoing total thyroidectomy: results of a preliminary study

**DOI:** 10.1186/1756-9966-29-77

**Published:** 2010-06-17

**Authors:** Guglielmo Ardito, Luca Revelli, Alma Boninsegna, Alessandro Sgambato, Francesca Moschella, Maria Cristina Marzola, Erica Giustozzi, Nicola Avenia, Mauro Castelli, Domenico Rubello

**Affiliations:** 1 Endocrine Surgery Unit, Department of General Surgery, Catholic University School of Medicine, Largo F.Vito 1, Roma 00168, Italy; 2 Institute of General Pathology, Catholic University School of Medicine, Largo F.Vito 1, Roma 00168, Italy; 3 Service of Nuclear Medicine & PET/CT Centre, Department of Imaging, Santa Maria della Misericordia Hospital, Via Tre Martiri 140, Rovigo 45100, Italy; 4 Department of Experimental Oncology, Regina Elena National Cancer Institute, Via delle Messi d'Oro 156, Roma 00158, Italy

## Abstract

**Background:**

Total thyroidectomy is the treatment of choice in the majority of thyroid malignancies, preventing the risk of reoperative surgery due to recurrences. In order to assess the usefulness of such an approach, expression levels of inflammatory and proliferative markers were evaluated immunohistochemically in non-lesional adjacent thyroid tissues from a group of patients who underwent total thyroidectomy for different thyroid diseases.

**Methods:**

Nineteen consecutive patients treated by total thyroidectomy for different thyroid diseases entered the study. IL-6Rb gp130 component of the IL-6 cytokine family members receptor complexes, STAT3 cytokine signalling transduction and transcription activation factor, p53 as tumour suppressor and CK19 cytokeratin as proliferation marker were analyzed in non-lesional thyroid tissues.

**Results:**

Gp 130 expression was detected in all tissue samples with a scattered distribution while STAT3 and p53 positivity was observed in 17 out of 19 patients with a prevailing cytoplasmic localization. Cytokeratin 19 positivity was found in patients with papillary carcinoma, in one case of follicular adenoma, 3 multinodular goiters and one Basedow disease.

**Conclusion:**

Based on the results of this preliminary study, it may be concluded that the presence of a persisting cytokine-mediated activation associated with cytoplasmic localization of p53 is frequently observed in different thyroid diseases. Such a process seems to occur in the thyroid gland as a whole. Moreover, STAT3 activation as well as mutant p53 are risk factors for the development of neoplastic diseases. Total thyroidectomy may be supported as an adequate therapeutic approach for all the patients in whom overexpression of cytokine-dependent markers is detected.

## Introduction

Thyroiditis includes a complex spectrum of pathologies in which inflammatory and autoimmune processes may be detected in coexistence with benign and malignant proliferative lesions [1].

Therefore total thyroidectomy is increasingly being considered as the treatment of choice,

preventing the risk of reoperation required for possible recurrences. The present study reports the expression of inflammatory and proliferative biological markers in non-lesional healthy thyroid tissue obtained from patients undergoing total thyroidectomy for various thyroid diseases. Our study tried to rationalise the usefulness of total thyroidectomy in the management of thyroiditis hypothesizing that in a chronic thyroid disease the associated inflammatory and/or autoimmune phenomenona may involve the whole gland and exert a modulatory effect with respect to carcinogenesis [2]. The IL-6 pro-inflammatory cytokine IL-6Rb gp130 component mediates high affinity binding of IL-6 to the IL-6Ra subunit, and constitutes the functional component of other IL-6 cytokine family members receptor complexes, such as Oncostatin M, Leukemia Inhibitory Factor and IL-11, through a wide array of inflammatory and immune responses [3]. Cytokine-dependent signalling activation involves the STAT proteins family as an important pathway to modulate different cell functions, where STAT3 plays a central role in transmitting signals from the membrane to the nucleus [4]. The tumour suppressor p53 senses multiplicity of cellular stresses, gets activated by post-translational mechanisms to induce cell-cycle arrest, senescence, or apoptosis and is a STAT3 functional regulator [5]. Constitutively active STAT3 is frequently expressed in a variety of human cancers and transformed cell lines associated to a mutated inactive p53 [6,7]. Thus, in this study, together with gp130, we analysed by immunohistochemistry the expression and intracellular localization of STAT3 and p53, to verify whether we could detect a cytoplasmic localization of the oncosuppressor protein indicative of its functional inactivation [8]. CK 19 cytokeratin which is expressed on epithelial tissue both in benign and malignant processes [9] was used as marker of epithelial tissue.

## Patients and Methods

Nineteen consecutive female patients who underwent total thyroidectomy for various thyroid diseases were investigated. Diseases included multinodular goiter (n = 10), follicular adenoma (n = 2), papillary carcinoma (n = 6) and Basedow disease [1]. Two patients with papillary carcinomas presented with concomitant Hashimoto disease or thyrotoxic goiter (Table 1). Mean age of the patients was 44 years (range 19-59) and disease duration ranged from 6 months to 25 years. Anti-thyroid antibodies were negative in all the patients.

**Table 1:** Results of the immunohistochemical staining on non-lesional tissue from 19 totally thyroidectomized patients.

**Patient n**.	Disease	STAT3	P53	CK19	Gp130
1	Multinodular goiter	N/C	N/C	neg	F

2	Papillary cancer	C	N/C	F	F

3	Follicular adenoma	N/C	N	ND	ND

4	Multinodular goiter	C	C	neg	Z

5	Multinodular goiter	C	C	neg	Z

6	Papillary cancer	N/C	C	Z	Z

7	Multinodular goiter	N/C	N/C	neg	Z

8	Multinodular goiter	neg	neg	neg	ND

9	Multinodular goiter	C	C	neg	F

10	Papillary cancer & Thyrotoxic goiter	N/C	C	F	F

11	Basedow disease	C	C	F	F

12	Papillary cancer	N/C	N/C	Z	Z

13	Multinodular goiter	N/C	N	neg	Z

14	Papillary c.	neg	C	Z	Z

15	Multinodular goiter	N/C	N/C	F	Z

16	Follicular adenoma	C	N	F	Z

17	Multinodular goiter	N/C	N/C	F	Z

18	Multinodular goiter	N/C	N/C	F	F

19	Papillary cancer & Hashimoto	C	C	F	Z

C = cytoplasmic; N = nuclear; F = focal; Z = zonal; ND = not determined; neg. = negative

Biopsy tissues used for immunohistochemical analyses were obtained from normal tissue adjacent to diseased areas. Samples were immediately frozen in liquid Nitrogen and stored at -80°C. On the day of analysis, tissue samples were gradually set to the temperature of -30°C for cryostat procedure. Seven sections were cut from each sample. The immunoperoxidase method was applied with Vector reagents utilizing the following primary antibodies: *a) *the anti-p53 polyclonal antibody CM-1 (Novocastra Laboratories Ltd) dilution 1:1000, *b) *the anti-STAT3 polyclonal antibody C-20 sc-482 clone (Santa Cruz Biotechnology) dilution 1:1000, *c) *the anti-CK19 monoclonal antibody b170 (Novocastra Laboratories Ltd) dilution 1:100, *d) *the anti-gp130 polyclonal antibody H-255 (Santa Cruz Biotechnology) dilution 1:250. The staining pattern was evaluated in epithelial cells both in terms of percentage of stained cells and staining intensity. In terms of percentage of stained cells, samples were classified as diffuse, zonal, focal and negative when the % of positive cells was >50%, between 10-50%, <10% and 0%, respectively. In terms of staining intensity, samples were subdivided into three categories: 1 + (low), 2 + (intermediate) and 3 + (high).

## Results

**Figure 1:**
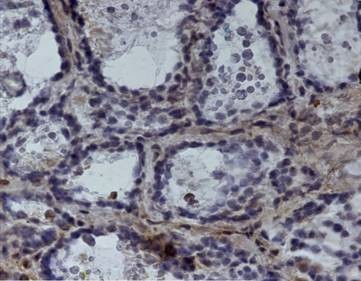
Himmunohistochemical positivity at p53

**Figure 2:**
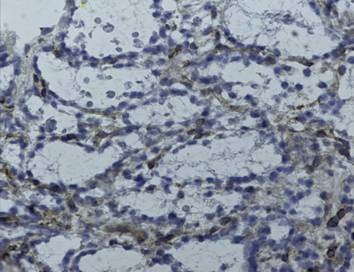
Himmunohistochemical positivity at gp130

The results of immunohistochemical analyses are shown in Table 1. Except for case number 8 (multinodular goiter) that was negative for both STAT3 and p53 expression, and case number 14 (papillary carcinoma) which was negative for STAT3, a diffuse pattern with an intermediate intensity in both nuclear and/or cytoplasmic localizations was observed in all the samples analyzed. An exclusive cytoplasmic localization of STAT3 was seen in 7 cases while a nuclear/cytoplasmic staining was detected in 10 cases. As for p53, three cases displayed an exclusive nuclear staining, 8 cases showed an exclusive cytoplasmic localization, 7 cases showed a nuclear/cytoplasmic positivity [Figure 1] and one case displayed no staining. gp130 staining was negative in two cases (3 and 8) while a zonal or focal membrane and cytoplasmic staining distribution of intermediate intensity (2+) was observed in most of the cases [Figure 2]. Cases 7, 15 and 19 showed an intense (3+) staining. Cytokeratin 19 (CK19) could not be determined in case 3, while 7 samples were negative, 8 showed a focal and 3 a zonal cytoplasmic distribution of intermediate intensity (2+).

## Discussion

The present work addresses the usefulness of total thyroidectomy based on biomarkers expression in apparently normal thyroid tissue in patients with various thyroid diseases. We evaluated gp130 expression as a constituent of receptor complexes common to a number of cytokines implicated in inflammatory and immune responses. Of these, Interleukin-6 (IL-6), a most important pleiotropic cytokine, plays a central role in immune regulation, inflammation, hematopoiesis, and oncogenesis. In our series, gp 130 expression was detected in all patients with a scattered distribution represented by groups of cells of variable size, confirming the involvement of cytokines signalling through the gp130 subunit. An earlier immunohistochemical study on the expression pattern of the IL-6 family members and their receptor subunits in normal prostate, benign prostatic hyperplasia, and prostatic carcinoma has suggested a role for this cytokine in both paracrine and autocrine regulation of proliferative processes [10]. In another study on oesophageal carcinoma it has been suggested that IL-6 may contribute to cancer progression in an autocrine or paracrine manner acting as an antiapoptotic factor [6]. As for STAT3 and p53 expression, both markers were found to be overexpressed in 17 out of the 19 patients studied with a prevailing cytoplasmic localization (in 5 cases we observed an exclusively cytoplasmic pattern). Although our series was relatively small and no robust statistical analysis could be performed, the data obtained did not show any significant differential pattern of distribution amongst tissues obtained from multinodular goiter, adenoma, autoimmune disease or papillary carcinoma. As previously mentioned, the transcription factor STAT3 is most important for the signal transduction of interleukin-6 and related cytokines. Upon stimulation cytoplasmic STAT3 is phosphorylated and translocates to the nucleus. When constitutively activated, STAT3 plays an important role in tumorigenesis, as shown in human breast cancer [5]. Wild-type p53 contributes to negatively regulate STAT3 phosphorylation. Thus, a mutant p53, as is the case for cytoplasmic p53, is also associated with constitutive STAT3 activation [7]. In the present study we did not investigate the STAT3 phosphorylation and the p53 mutational status as our aim was to evaluate their subcellular localization in apparently normal thyroid tissue and to verify whether differences exist amongst different thyroid diseases. The results are suggestive of an ongoing modulation mechanism, where an increased p53 expression level is observed with a main cytoplasmic localization, going along with an almost equivalent localization pattern for STAT3.

## Conclusion

On the basis of the present data, it may be concluded that activation of STA3 signalling is a frequent event in thyroid diseases, being observed in normal thyroid tissue adjacent to different types of thyroid diseases. Such a process seems to involve the whole thyroid gland. Since a constitutively active STAT3, associated to cytoplasmic accumulation of p53, has been reported to represent a risk factor for tumor development [11], total thyroidectomy may be supported as an adequate therapeutic choice in cases where such alterations are detected.

## Competing interests

The authors declare that they have no competing interests.

## Authors' contributions

GA performed thyroid surgery, participated in study design and coordination. LR participated to perform thyroid surgery, participated in the sequence alignment and drafted the manuscript. AB performed immunohistochemical studies, participated in study design and coordination. AS participated to perform immunohistochemical studies, participated in study design and coordination FA participated to perform thyroid surgery, participated in the sequence alignment and drafted the manuscript. MCM participated in the sequence alignment and drafted the manuscript. EG participated to perform thyroid surgery, participated in the sequence alignment and drafted the manuscript. NA performed thyroid surgery. MC participated in the sequence alignment and drafted the manuscript. DR participated in the sequence alignment and drafted the manuscript. All authors read and approved the final manuscript.
